# Laboratory and Field Evaluation of the Crystal VC-O1 Cholera Rapid Diagnostic Test

**DOI:** 10.4269/ajtmh.20-1280

**Published:** 2021-04-05

**Authors:** Amanda K. Debes, Kelsey N. Murt, Ethel Waswa, Gerald Githinji, Mamo Umuro, Caroline Mbogori, Mellisa Roskosky, Malathi Ram, Allison Shaffer, David A. Sack, Waqo Boru

**Affiliations:** 1 Department of International Health, Johns Hopkins School of Public Health, Baltimore, Maryland;; 2 Ministry of Health, Nairobi, Kenya;; 3 Field Epidemiology and Laboratory Training Program, Nairobi, Kenya

## Abstract

Cholera is a severe acute, highly transmissible diarrheal disease which affects many low- and middle-income countries. Outbreaks of cholera are confirmed using microbiological culture, and additional cases during the outbreak are generally identified based on clinical case definitions, rather than laboratory confirmation. Many low-resource areas where cholera occurs lack the capacity to perform culture in an expeditious manner. A simple, reliable, and low-cost rapid diagnostic test (RDT) would improve identification of cases allowing rapid response to outbreaks. Several commercial RDTs are available for cholera testing with two lines to detect either serotypes O1 and O139; however, issues with sensitivity and specificity have not been optimal with these bivalent tests. Here, we report an evaluation of a new commercially available cholera dipstick test which detects only serotype O1. In both laboratory and field studies in Kenya, we demonstrate high sensitivity (97.5%), specificity (100%), and positive predictive value (100%) of this new RDT targeting only serogroup O1. This is the first field evaluation for the new Crystal VC-O1 RDT; however, with these high-performance metrics, this RDT could significantly improve cholera outbreak detection and improve surveillance for better understanding of cholera disease burden.

## INTRODUCTION

Cholera is a diarrheal disease caused by the bacterium *Vibrio cholerae*. It typically occurs in low- and middle-income countries in settings that lack access to clean water and proper hygiene and sanitation. Although confirmation of the etiologic agent is not necessary for treatment of the disease, confirmation of the diagnosis is vital to identify and respond to outbreaks and to better understand the epidemiology of the disease. Unfortunately, cholera confirmation is often challenging because the disease tends to occur in resource-constrained settings; these areas often lack sophisticated microbiology laboratories with skilled personnel. When these laboratories are available, the classical culture method requires serological and biochemical tests which require several days before a report can be issued. Increasingly, a PCR is being used to confirm the diagnosis^1^ and can be useful for certain situations, but this assay has not been widely used because of lack of PCR equipment, reagents, and trained staff.

In 2017, the Global Task Force on Cholera Control (GTFCC) led by the WHO announced their “Roadmap to End Cholera by 2030,” and a key element of the road map is the ability to rapidly respond to outbreaks.^2^ A reliable rapid diagnostic test (RDT) that could be used in low-resource settings would greatly facilitate this goal. Several commercial RDTs are available and are being used during outbreaks or for surveillance.^1,3–8^ These tests are portable and can be performed in nearly any environment. However, the currently available tests have demonstrated a wide range of sensitivities and specificities and their accuracy and reliability can vary based on the user and situation.^9,10^ Because of the lack of confidence in current cholera RDTs, public health officials have been reluctant to declare an outbreak based solely on results of an RDT; this can result in delays in response to an outbreak.^11^

Crystal VC is an RDT (Arkray Healthcare Pvt. Ltd, Gujarat, India) that tests for both serogroups O1 and O139. However, specificity for this test has been problematic, requiring confirmation of the diagnosis by culture because false-positive reactions,^12^ especially for serotype O139,^6,13–16^ have occurred. Because of this issue, the Centers for Disease Control advises, “it is recommended that fecal specimens that test positive for *V. cholerae* O1 and/or O139 by the Crystal^®^ VC dipstick always be confirmed using traditional culture-based methods suitable for the isolation and identification of *V. cholerae.*”^17^ Theoretically, the ability to detect serotype O139 could be of benefit; however, cholera due to *V. cholerae* serotype O139 has never been detected in Africa and is now very rarely identified in the South Asia and China during the last two decades.^18–20^

A new version of the RDT was recently developed and is marketed as Crystal VC O1 (Arkray Healthcare Pvt. Ltd). This new version tests solely for *V. cholerae* O1 with the intention to improve the accuracy of the assay for serotype O1. Thus, this RDT eliminates concerns for false O139 positives with the potential to increase specificity. If a cholera RDT is developed and demonstrates high sensitivity and specificity, this will greatly change the landscape of cholera detection and control as well as our understanding of true burden of disease. In this study, we conducted laboratory-based testing of a Crystal VC O1 in comparison to the original Crystal VC. Subsequently, we conducted a field evaluation of the test during outbreaks in Kenya from 2018 to 2019 to evaluate the RDT for sensitivity and specificity in comparison to both standard microbiological culture and PCR.

## METHODS

### Ethics.

Ethical approval to conduct the study was obtained from the AMREF Health Africa Ethics and Scientific Review Committee (ESRC P552/2018) and the Johns Hopkins Bloomberg School of Public Health Institutional Review Board (IRB00009067). Outbreaks were responded to according to Ministry of Health Protocol, by the Ministry of Health personnel. Specimens were collected through the Ministry of Health’s disease surveillance program. Patients suspected of having cholera gave informed consent for participation in the study (including parental permission/child assent) before enrollment in the study. Personal identifiers were removed upon enrollment.

### Study site.

**Figure 1.**
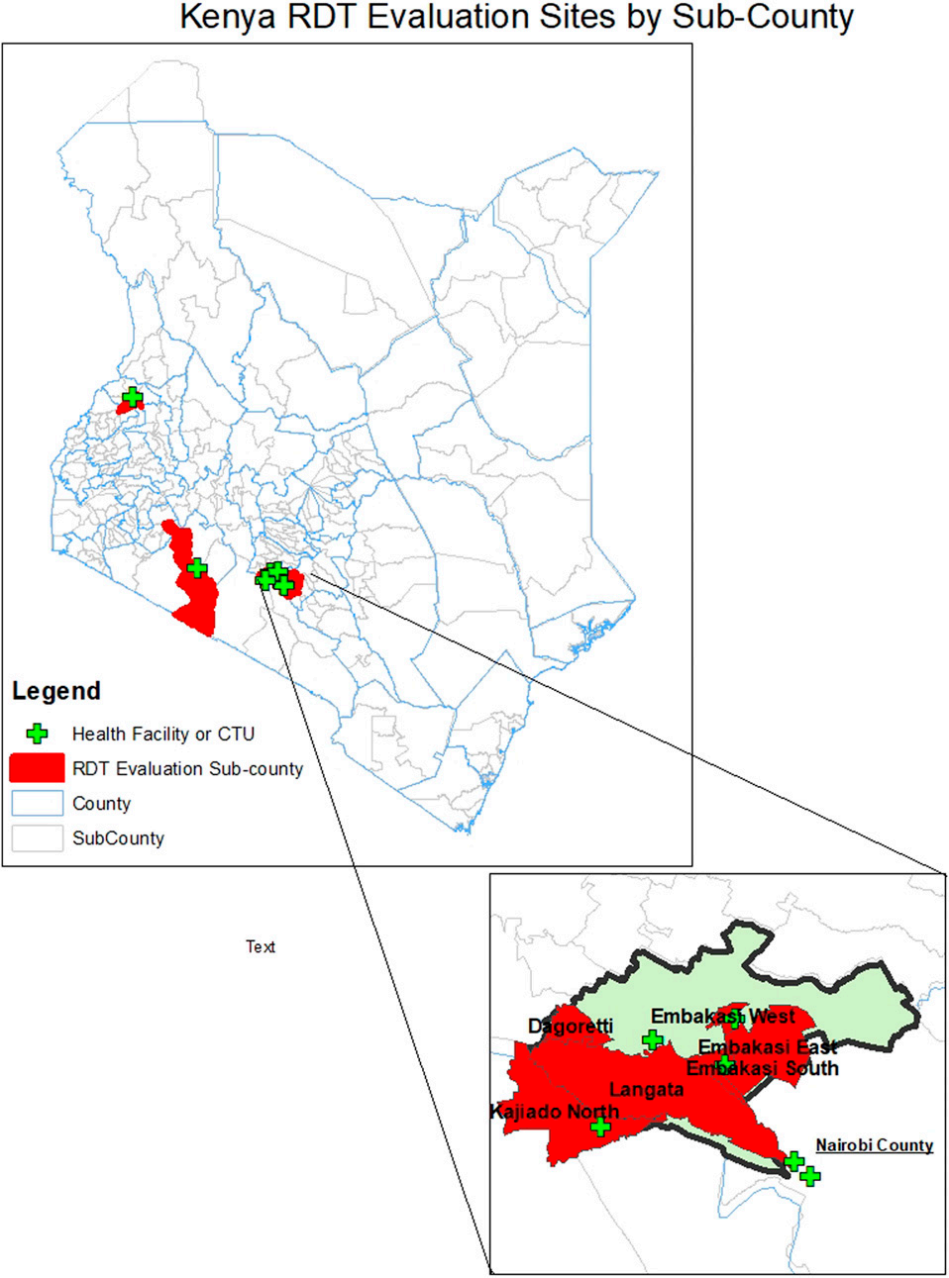
Map of rapid diagnostic test (RDT) evaluation sites.

Crystal VC-O1 was evaluated, in comparison to Crystal VC, for specificity and cross-reactivity in the Enteric Laboratory of the International Health Department of the Johns Hopkins Bloomberg School of Public Health, Baltimore, MD. Field evaluations were conducted in the Republic of Kenya where cholera is endemic and has been reported regularly since 1971.^21^ A cholera outbreak beginning in December 2014^22^ has challenged the Kenyan health system and continued through the evaluation of this RDT. Stool samples were collected from December 2018 to May 2019 at cholera treatment centers (CTCs) and healthcare facilities throughout Kenya (Figure 1). Laboratory testing was completed at the National Public Health Laboratory, Ministry of Health, Kenya.

### Study Procedures.

#### Specificity and cross-reactivity testing, Baltimore, MD.

Stool was collected from a healthy individual at three different time points and stored at 4°C until testing. The stool was suspended in sterile, nuclease-free distilled water mixed by vortexing until getting a homogenous mixture at a ratio of 1 g of stool to 3 mL of water.

Strains of *Vibrio* were cultured to create test combinations of *Vibrio* strains in the spiked healthy stool. The strains used in this study included strains of *Vibrio cholerae* O1 Ogawa, *Vibrio cholerae* O1 Inaba, *V. cholerae* O139, nontoxigenic *V. cholerae*, and *V. cholerae* non-O1, non-0139 (Supplemental Table 1). Frozen stocks were revived via direct streaking on thiosulfate citrate bile salt sucrose (TCBS; Becton Dickinson and Co., Franklin Lakes, NJ) agar followed by overnight incubation at 37°C. A sterile cotton swab was used to touch 10 colonies from the plate and resuspend in 3 mL (mL) of PBS. And, 0.1 mL of the suspension is used to subculture lawns of the bacteria on Luria agar overnight at 37°C. The bacterial lawn is harvested with sterile PBS. The optical density of the bacterial suspension was determined by measurement at 600 nm, and the bacterial–stool mixture was diluted to a final concentration of approximately 1 × 10^8^ bacteria per milliliter. The number of colony-forming units (CFU) in the inoculum was confirmed by titrating and plating on Luria agar plates.^23^ Bacterial–stool suspensions were created for nontoxigenic *V. cholerae*, *V. cholerae* non-O1, non-O139, *V. cholerae* O1 Ogawa, *V. cholerae* O1 Inaba, and *V. cholerae* O139 bacteria.

Specificity testing was performed by testing combinations of strain-spiked stool samples to validate specificity to *V. cholerae* O1. Testing was performed in triplicate with Crystal VC and Crystal VC-O1 RDTs. Cross-reactivity testing was performed using one spiked sample per specified combination. Before testing, each sample was vortexed for an additional 30 seconds. The samples were tested using the RDTs following the manufacturers’ instructions for testing fecal samples.^24^

#### Field evaluation study procedures, Kenya.

Patients of any age who presented to the health facility seeking care for acute watery diarrhea were eligible for inclusion in the study. No other inclusion or exclusion criteria were applied. A fresh stool sample, and basic clinical and demographic characteristics were collected from patients who consented to participate. Information collected included, but was not limited to, location of the health facility, age, gender, and dehydration status (Table 3). Standard medical care was provided by the healthcare facility, and participation in the study did not affect the care provided. Fresh stool specimens were immediately tested using the Crystal VC O1 RDT according to manufacturers’ instructions. In brief, approximately two drops of liquid stool were transferred to the reagent vial included in the Crystal VC/Crystal VC-O1 kit. For solid stools, a small portion of stool was collected with the sampling stick and then transferred to the reagent vial and shaken to mix. From the mixed reagent vial, four drops were then transferred to the provided test tube. A dipstick was removed from its sealed packaging and then placed into the liquid stool mixture such that only the absorbent tip was immersed. The strip was removed after 15 and before 30 minutes and read by two independent technicians, and results were recorded separately. The dipsticks were photographed to preserve the appearance of the dipstick. A positive result appears as two pink lines on the dipstick, the upper line being the control band and the lower line being the band specific to serogroup O1.

An aliquot of the stool samples was preserved used for standard microbiological methods. Prepackaged Cary-Blair tubes were labeled with the specimen ID, the supplied swab was inserted into the stool sample and subsequently used to inoculate the media. All specimens were cultured within 3 days of collection. The microbiology laboratory recorded the results of the microbiological culture tests independent of the results of the rapid test.

In addition to the RDT and microbiological culture, two drops of the stool specimen were spotted and dried on a Whatman 903 Protein Saver Card (10534612 903; Sigma-Aldrich, St. Louis, MO; catalog no. WHA10534612). The filter paper specimen was then stored in a plastic bag for PCR testing for *V. cholerae* at Johns Hopkins University.^1,25^ The laboratories were blind to the results of tests completed in separate laboratories.

### Laboratory methods.

#### Microbiological stool culture*.*

Swabs in specimen-labeled Cary-Blair tubes were used to streak TCBS plates according to published protocol.^26^

#### Multiplex PCR*.*

Stool samples preserved on Whatman 903 Protein Saver cards were shipped to the laboratory at Johns Hopkins University. DNA was extracted from filter papers according to previously published methods.^1^ In brief, the stool-spotted filter paper was excised and rehydrated in 1 mL of sterile 1X PBS for 10 minutes, centrifuged (14,000 × *g* for 2 minutes), and the supernatant discarded. Then, 1 mL of sterile 1X PBS was added to the sample tube and immediately centrifuged, and the supernatant was discarded. Subsequently, 140 µL of 2% (w/v) Chelex-100 (Bio-Rad, Hercules, CA) was added to each sample. And then, 50 µL of sterile water was added to each sample, followed by heating at 100°C for 8 minutes. The samples were then centrifuged, and the supernatant was transferred to a clean microcentrifuge tube and stored at −20°C until PCR processing. Conventional PCR was performed targeting the ompW and cholera toxin A gene (ctxA) genes, as previous described.^25^ The Nandi multiplex targets 1) the outer membrane protein (OmpW), a gene conserved in the *V. cholerae* genome; and 2) the ctxA. The PCR was performed, as published, using Terra PCR Direct Polymerase (Takara, Mountain View, CA).

#### Statistical methods.

A true cholera case was defined as a clinically suspected case with either a positive culture or PCR result for *V. cholerae* O1. A true-negative case was defined as a suspected case that was negative by culture and PCR for *V. cholerae* O1. The sensitivity, specificity, positive predictive values (PPVs) and negative predictive values (NPVs) were calculated using the Stata command diagti for both laboratory evaluations and field evaluations. The analysis was conducted in Stata 16 (Stata Corporation, College Station, TX). The kappa coefficient was calculated in Stata using the kap command to estimate the agreement between the gold standards (culture and/or PCR) and the RDT. Chi-square and *T*-test analyses were used evaluate clinical characteristics of study participants.

## RESULTS

### Laboratory evaluation.

Table 1 demonstrates the sensitivity of both the original Crystal VC RDT and the new Crystal VC O1 RDT. In three evaluations of stool spiked with nontoxigenic O1, non-O1/non-O139, or O139 *Vibrio* strains, both RDTs accurately displayed positive results for samples spiked with O1 strains, and negative results for the non-O1/non-O139 strains. For the O139 spiked stools, the Crystal VC O1 showed a negative result, whereas the Crystal VC RDT was positive for O139.

**Table 1:** Laboratory evaluation of Crystal VC and Crystal VC O1

Serotype	Trial	Crystal VC O1	Crystal VC	
		O1 line	O1 line	O139 line
Nontoxigenic *V. cholerae O1*	1	**+**	**+**	**–**
	2	**+**	**+**	**–**
	3	**+**	**+**	**–**
*V. cholerae (non-O1* and *non-O139)*	1	**–**	**–**	**–**
	2	**–**	**–**	**–**
	3	**–**	**–**	**–**
*V. cholerae O139*	1	**–**	**–**	**+**
	2	**–**	**–**	**+**
	3	**–**	**–**	**+**

*V. cholerae = Vibrio cholerae.* Results from stool spiking of various strains of *V. cholerae* for both Crystal VC and Crystal VC O1 rapid tests. Three separate stools were used for three independent assays, and stools were spiked with a single strain for each assay.

As shown in Table 2, the Crystal VC–O1 RDT yielded a positive result with both the Inaba and Ogawa strains when combined with different *Vibrio* strains (non-O1/non-O139, nontoxigenic O1, and O139).These results demonstrate ability of the tests to accurately identify *V. cholerae* O1 without cross-reactivity from other *Vibrio* species that may be present in the sample. Similarly, when evaluated for cross-reactivity, the Crystal VC RDT accurately showed a positive result for O1 for all stool aliquots containing *V. cholerae O1* and accurately showed a positive result for O139 for all stool aliquots containing *V. cholerae O139*. No false-positive results were observed for either test.

**Table 2:** Laboratory evaluation of cross-reactivity

*Vibrio cholerae* serotype		Crystal VC O1	Crystal VC	
Strain 1	Strain 2	O1 line	O1 line	O139 line
O1 Inaba	*O1* (nontoxigenic)	**+**	**+**	**–**
O1 Inaba	*non-O1* and *non-O139*	**+**	**+**	**–**
O1 Ogawa	*O1* (nontoxigenic)	**+**	**+**	**–**
O1 Ogawa	*non-O1* and *non-O139*	**+**	**+**	**–**
O139	*O1* (nontoxigenic)	**+**	**+**	**+**
O139	2*non-O1* and *non-O139*	**–**	**–**	**+**

Results from stool spiking of two strains of *Vibrio cholerae* to evaluate cross-reactivity of Crystal VC and Crystal VC O1 rapid tests.

### Field evaluation.

**Figure 2.**
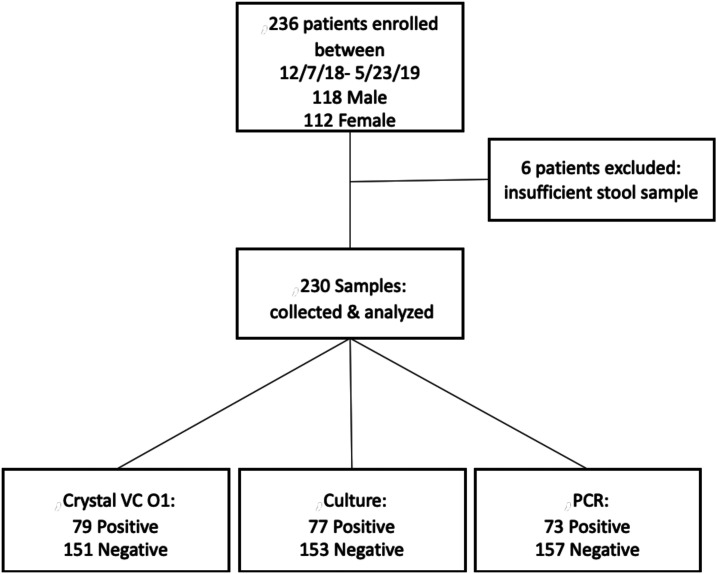
Study flow chart.

From December 2018 to May 2019, a total of 236 patients were enrolled, from which 230 had specimens suitable for inclusions in the study. The participants presented at health facilities in nine subcounties, representing five counties: 3-Mavoko (Machakos); 10-Dagoretti North, 17-Embakasi East, 6-Embakasi South, 93-Embakasi West, 5-Langata, (Nairobi); 11-Kaijado North (Kaijado); 36-Narok South (Narok); and 49-Kimini (Trans-Nzoia). There were 112 female and 118 male participants that ranged in age from infant to 64 years old, with a mean age of 25. The demographic and clinical characteristics of the participants in the study is shown in Table 3. A flow chart summarizing the results of the different tests with these specimens is shown on Figure 2.

Of the 230 stool samples obtained for this analysis, 77 were positive for *V. cholerae* by culture methods, 79 were positive by RDT and 73 were positive by PCR. All culture-positive samples were also RDT positive. Two samples were RDT positive and PCR positive but culture negative. The comparison of the culture and PCR results with RDT, sensitivity, specificity, and positive and negative predictive values is shown in Table 4. As noted, the two specimens that were culture negative by culture but RDT positive were found positive by PCR, and thus considered positive. Two additional specimens were positive by PCR but negative by culture and RDT. Eight specimens were negative by PCR but positive by culture and RDT.

**Table 3:** Participant demographics and clinical status

Variable		RDT positive (*n* = 79)	RDT negative (*n* = 148)	*P*-value
Gender (%)	Male	50 (42.4)	68 (57.6)	0.013
	Female	29 (25.9)	83 (74.1)	
Mean age (years) (±SD)		28.1 (14.86)	24.5 (14.6)	0.1276
Age category (years)	0–4 (%)	7 (25)	21 (75)	0.499
	5–14	6 (34.6)	13 (68.4)	
	≥ 15	66 (36.1)	117 (63.9)	
Type of diarrhea	Bloody	0 (0)	1 (100)	< 0.001
	Mucus	15 (16.1)	78 (83.9)	
	Watery	64 (47.4)	71 (52.6)	
Dehydration status (%)	Plan A (none)	1 (1.2)	81 (98.7)	< 0.001
	Plan B (moderate)	36 (39.6)	55 (60.4)	
	Plan C (severe)	42 (73.7)	15 (26.3)	
IV Rehydration (%)	No	0 (0)	82(100)	< 0.001
	Yes	79 (54.1)	67(45.9)	

**Table 4:** Sensitivities and specificities of Crystal VC O1 compared with culture or PCR in a field evaluation

	Culture or PCR positive	Culture or PCR negative	Culture positive	Culture negative	PCR positive	PCR negative
RDT positive	79	0	77	2	71	8
RDT negative	2	149	0	151	2	149
Total	81	149	77	153	79	151
Sensitivity	97.5% (91.4–99.7)		–	–	–	–
Specificity	100% (97.6–100)		–	–	–	–
Positive predictive value	100% (95.4–100)		–	–	–	–
Negative predictive value	98.7% (95.0–99.7)		–	–	–	–

The RDT was positive for 79 of 81 samples found positive by either test, as shown in Table 4. Applying this definition to our results, the sensitivity of the RDT test was 97.5%, and specificity was 100%, the positive predictive value was 100%, and the negative predictive value was 98.7%. There were no false-positive results using the RDT. The overall agreement between Crystal VC-O1 and culture was kappa = 0.9806, and between Crystal VC-O1 and PCR was Kappa = 0.9018.

## DISCUSSION

Laboratory-based evaluations for sensitivity and cross-reactivity of Crystal VC O1 demonstrated high sensitivity and specificity. There was no evidence of interference to the RDT performance for either O1 Inaba or O1 Ogawa strains when evaluated in the presence of other *Vibrio* strains. Experiments evaluating different strains reproducibly confirmed that the RDT accurately yielded a positive result for O1 when tested with a nontoxigenic O1 strain and a negative result when tested with a non-O1/non-O139 strain. In addition, the Crystal VC test yielded a positive result for O139 for stools spiked with O139.

The current price of the kit is approximately $2 USD, similar to the original Crystal VC RDT. It is of similar ease of use as the original Crystal VC RDT, with comparisons to the use of a pregnancy test. The aspects of RDT use which remain critical include diluting the specimen in the sample buffer, ensuring the RDT is not submerged past the line of demarcation on the RDT and ensuring the test is read 15–30 minutes from the time it was started. The RDT kit includes all necessary supplies for the RDT test itself, but sample collection cups for the patient must be supplied. Furthermore, if testing after enrichment in alkaline peptone water (APW) is desired, the APW will be needed.^27^

The field evaluation corroborated the laboratory evaluation, demonstrating that Crystal VC-O1 yielded high sensitivity (%) and specificity when used to identify *V. cholerae* O1–positive patients presenting with watery diarrhea. When comparing the results of the three cholera detection methods used (RDT, culture, and PCR), most of the samples in the field evaluation were concordant. Among the few samples in which there were discordant results, two samples were positive by RDT and PCR but negative by culture. Because the samples were found to be positive by PCR, which is more sensitive, suggesting that the RDT result was correct and the culture was falsely negative. In addition, two samples were positive only by PCR but negative by culture and RDTs. Because PCR is more sensitive, it is likely that these samples contained a very low concentration of *V. cholerae* and/or that the participant had taken antibiotics before reporting to the health facility, causing the *V. cholerae* to be undetectable by the other two tests. Finally, there were eight samples that were positive by culture and negative by PCR; this was unexpected, given the high sensitivity of the PCR. Four of these samples came from a single cluster of cases. To further investigate these results, we will conduct additional studies to investigate this finding. This is likely due to an inadequate amount of stool placed on the filter paper. In all eight of these samples, the RDT and culture results were in agreement, so the RDT results were considered accurate in accordance with our definition of a true cholera case. Although additional studies are needed to confirm these results with this new RDT in different settings, our study team responded to outbreaks in different regions of Kenya and were able to use the RDT in the various field settings, maintaining its high performance. Using the manufacturers’ instructions, the Crystal VC-O1 RDT meets the minimum and the desired performance recommendations set forth by the GTFCC for cholera RDTs.^28^

Studies evaluating the original Crystal VC in comparison to culture and PCR report sensitivities in the range from 65.6 to 99% and specificities from 49.2% to 95.7%.^5,6,9,12,29,30^ This field evaluation found sensitivity and specificities at the higher end of this range for both sensitivity and, particularly, specificity. In most of the previous studies, direct specimen testing via RDTs shows higher sensitivity and lower specificity, whereas in our Crystal VC-O1 evaluation, we found extremely high sensitivity and specificity. In fact, the Crystal VC-O1 evaluation reported higher specificities than those in any of the previous studies evaluating Crystal VC. Given the historical lack of specificity with using Crystal VC directly, the performance metrics found in this evaluation may significantly impact cholera RDT use.

Patients with more severe disease tended to have a higher proportion of positive tests; 73.7% of severely dehydrated patients were RDT positive. However, some patients with moderate to severe dehydration reporting watery diarrhea were RDT and culture negative, suggesting that there are other pathogens able to cause moderate to severe diarrhea even during an outbreak. We did not search for other pathogens in this study, but a likely pathogen is enterotoxigenic *Escherichia coli*.

There were several limitations in this study. First, there were samples that were positive by culture but negative by PCR. It is suspected that insufficient amount of stool was spotted on the filter paper for preservation. The specimens were not enriched in APW and tested via RDTs because of preservation of the RDT for direct assessment. Such enrichment would have facilitated a full comparison of the RDTs performance metrics in comparison to those required by the Target Product Profile (TPP).^28^ In addition, we did not explore the 151 negative specimens to understand the pathogens presenting with similar clinical indications. Finally, this evaluation was conducted only within Kenya and for the results to be generalizable to other cholera prone countries, the evaluation must be reproduced in additional settings.

In conclusion, the new Crystal VC-O1 RDT, with a single line for serogroup O1, performed with high sensitivity and high specificity in this study. Importantly, we did not find any false-positive results. If this is confirmed in other studies, this suggests that an outbreak could be confirmed based on a positive Crystal VC-O1 RDT, particularly if specimens from multiple patients are positive. The impact that a cholera RDT with such high sensitivity and specificity can have on cholera surveillance and control is vast; not only would we have an improved understanding of the true cholera burden but also such an RDT would eliminate many of the laboratory challenges surrounding cholera confirmation, expedite outbreak detection and response, improving disease-monitoring methods in endemic and high-risk areas.

## Supplemental tables

Supplemental materials
