# Supplementary material for: Laboratory and Field Evaluation of the Crystal VC-O1 Cholera Rapid Diagnostic Test
Source: Am J Trop Med Hyg. 2021 Apr 5;104(6):2017–23. doi: 10.4269/ajtmh.20-1280 (PMC8176501; doi:10.4269/ajtmh.20-1280)
Supplement: Supplementary file 1 [file tpmd201280.SD1.docx]

| **Supplemental Table 1: Strain information for cross-reactivity in lab assays** | | | |
| --- | --- | --- | --- |
| ***Vibrio Cholerae* Serotype** | **Strain ID** | **Location** | **Year** |
| O1 Inaba | **1827 (NHCM-0059)** | **Bangladesh** | **2012** |
| O1 Ogawa | **1706 (NHCC-090)** | **Bangladesh** | **2011** |
| O139 | **ATCC51394** | **India** |  |
| O1 non- toxogenic Inaba | **CAH184*** | **Bangladesh** |  |
| non-O1, non-O139 | **1717 (EC-0060)** | **Bangladesh** | **2011** |
| **Hauke CA, Taylor RK, 2017. Production of putative enhanced oral cholera vaccine strains that express toxin-coregulated pilus. PLoS One 12: e0175170* | | | |
